# Upper limb soft robotic wearable devices: a systematic review

**DOI:** 10.1186/s12984-022-01065-9

**Published:** 2022-08-10

**Authors:** Elena Bardi, Marta Gandolla, Francesco Braghin, Ferruccio Resta, Alessandra L. G. Pedrocchi, Emilia Ambrosini

**Affiliations:** 1 Department of Mechanical Engineering, Politecnico di Milano, Milano, Italy; 2 Department of Electronics, Information and Bioengineering, Politecnico di Milano, Milano, Italy

**Keywords:** Assistive technology, Exoskeletons, Exosuits, Rehabilitation robotics, Soft robotics, Upper limb

## Abstract

**Introduction:**

Soft robotic wearable devices, referred to as exosuits, can be a valid alternative to rigid exoskeletons when it comes to daily upper limb support. Indeed, their inherent flexibility improves comfort, usability, and portability while not constraining the user’s natural degrees of freedom. This review is meant to guide the reader in understanding the current approaches across all design and production steps that might be exploited when developing an upper limb robotic exosuit.

**Methods:**

The literature research regarding such devices was conducted in PubMed, Scopus, and Web of Science. The investigated features are the intended scenario, type of actuation, supported degrees of freedom, low-level control, high-level control with a focus on intention detection, technology readiness level, and type of experiments conducted to evaluate the device.

**Results:**

A total of 105 articles were collected, describing 69 different devices. Devices were grouped according to their actuation type. More than 80% of devices are meant either for rehabilitation, assistance, or both. The most exploited actuation types are pneumatic (52%) and DC motors with cable transmission (29%). Most devices actuate 1 (56%) or 2 (28%) degrees of freedom, and the most targeted joints are the elbow and the shoulder. Intention detection strategies are implemented in 33% of the suits and include the use of switches and buttons, IMUs, stretch and bending sensors, EMG and EEG measurements. Most devices (75%) score a technology readiness level of 4 or 5.

**Conclusion:**

Although few devices can be considered ready to reach the market, exosuits show very high potential for the assistance of daily activities. Clinical trials exploiting shared evaluation metrics are needed to assess the effectiveness of upper limb exosuits on target users.

## Background

Neuromuscular diseases (e.g., stroke, spinal cord injury, muscular dystrophy, etc.) and neurodegenerative diseases (e.g., multiple sclerosis, amyotrophic lateral sclerosis, etc.) can lead to severe motor impairment. On the one hand, this requires the patients to undertake a rehabilitation path to mitigate negative effects and improve motor functions and their general state of health. On the other hand, patients might become dependent on long-term care assistance for activities of daily living (ADLs).

Disabilities of the upper limb have a strong impact on the subject’s quality of life since they affect the possibility to independently perform basic activities [1, 2]. In this context, wearable rehabilitative and assistive devices, such as exoskeletons, may play an important role [3]. Exoskeletons are composed of rigid links, that are attached to the user’s limbs, and actuators, which exert torques at the joint level [4]. The main scenarios for which exoskeletons have been developed are: (i) motor rehabilitation of impaired limbs (rehabilitation scenario), (ii) assistance of subjects with disability with ADLs (assistive scenario), (iii) motor augmentation of healthy subjects in contexts such as factory work, military applications or sport (augmentation scenario).

In the rehabilitation scenario, wearable robots support the therapist in providing rehabilitative exercises. The advantage they bring with respect to traditional therapy lies in the higher number of repetitions that can be provided in a session, the possibility of objectively quantifying the subject’s performance, the relief of the therapist’s physical burden, and the possibility to monitor the patient’s involvement in the training. This makes it possible to increase the dose, personalize the intensity of the training, and stimulate the participation of the subject, which are all key factors in motor re-learning [5–7].

In the assistance scenario, wearable robots are meant to support movements typical of ADLs, such as drinking, eating, reaching, and personal hygiene [8, 9]. The use of assistive devices could help the user gain back part of his/her independence and facilitate participation, which is fundamental from a psychological and social point of view.

In the augmentation scenario, wearable robots provide high torques to improve the subject’s capabilities beyond the physiological level or to share and redistribute the load applied on the limbs. The main goal is to prevent musculoskeletal diseases typical of fatiguing and repetitive work and to reduce the metabolic cost [10].

A recent review written by Xiloyannis and colleagues [11] features a taxonomy useful to classify the different types of wearable robots to assist or augment the user’s movements. The first branching of their taxonomy classifies the devices between those that rely on a rigid frame to exert torques, referred to as “rigid exoskeletons”, and those that do not, referred to as “soft robotic suits”.

Although rigid exoskeletons can provide good trajectory tracking and can exert high torques, which are borne by the exoskeletal structure, they present numerous disadvantages: (i) they are heavy and bulky, which increases the inertia of the system and the metabolic cost of wearing; (ii) they are expensive; (iii) the rigid links constrain the natural degrees of freedom of the human joints and require careful alignment, which is time-consuming; (iv) even the smallest misalignment leads the exoskeleton to interfere with the physiological movements of the limb; (v) they have a low aesthetic quality. All these drawbacks prevent rigid exoskeletons to be widely adopted outside the clinical environment and to be used for home rehabilitation or daily assistance [12].

Therefore, soft robotic devices have been recently proposed as a valid alternative. Robotic suits are inherently compliant thanks to the lack of rigid links and the use of soft materials, such as fabric or soft polymers, as an interface with the subject’s limbs [13]. The use of such materials brings several advantages: (i) it supports the wearer’s movements without over-constraining the joints, thus maintaining their mobility and flexibility; (ii) precise joint alignment is not required, reducing the time needed to wear the device; (iii) it improves the comfort of wear and ease of donning and doffing, thus improving usability; (iv) it reduces the overall weight of the device, as well as the encumbrance, thus improving the portability; (v) it reduces the cost. These characteristics make this relatively new technology quite promising in delivering rehabilitation and providing assistance outside the clinical context.

However, despite the numerous advantages listed, robotic suits present some challenges that require further research. In particular, they act more as an external muscle, rather than an external skeleton [11]. This means that the actuation relies on the skeletal structure of the user, preventing the application of high torques, which may hurt the wearer. Moreover, their intrinsic compliance sacrifices the accuracy of the movements and the magnitude of the assistance, thus making the control quite complex. Indeed, the sleeve may slide, influencing the data collected by the sensors and making the force transmission unreliable [14, 15]. The shear forces acting on the skin could be increased as well. In addition, when it comes to upper limb assistance, a further challenge of soft robotic devices is represented by the control of upper limb movements. In fact, lower limb devices usually implement control strategies that rely upon the cyclicality of walking. In upper limb devices, instead, the number of dynamic tasks to be implemented, the unpredictable interaction with the environment, and the complexity of the biomechanics make their control a more complex operation [16].

### Objective

In view of the growing research interest in the field, and given that no device has reached the market yet, we provide a complete and systematic literature review of soft robotic devices for upper limb assistance.

Xiloyannis and colleagues [11] provide an insightful narrative review on the modes of actuation, the physical human-robot interfaces, and the intention-detection strategies of some of the state-of-the-art soft devices, both for the upper and the lower limbs. However, they do not provide a complete list of all the devices. Another recent review [17] focuses only on the description of the different types of actuators for soft robotic devices.

The aim of this review is instead to provide a broad picture of the state of the art to help researchers that approach this field. Indeed, it investigates the possibilities for the application scenario, the actuation and the actuated joints, the design approaches, the intention detection strategies, and the validation experiments that might be exploited in the process of developing an upper limb robotic suit.

## Methods

### Search methods

We run a systematic review using the keywords in the electronic database search shown in Table 1. Keywords are subdivided into three categories: (1) the type of device, (2) the attributes of interest, and (3) the body section the devices interact with. The keywords were combined using Boolean operators (AND/OR) as follows: (1 OR 2 OR ... 7) AND (8 OR 9 OR ... 15) AND (16 OR 17 OR ... 22).

**Table 1:** List of the keywords used for the electronic search

Device	Attributes	Body section
1. Exoskeleton	8. Soft	16. Upper limb
2. Robot	9. Flexible	17. Arm
3. Exosuit	10. Wearable	18. Shoulder
4. Exosleeve	11. Inflatable	19. Elbow
5. Sleeve	12. Cable-driven	20. Forearm
6. Orthosis	13. Pneumatic	21. Wrist
7. Suit	14. Fabric-based	22. Upper extremity
	15. Portable	

We restricted the research among papers written in English and published between 2000 and 2020. We searched the following bibliographic electronic databases: PubMed, Scopus, Web of Science. Furthermore, we performed backward and forward reference searching on the relevant articles identified from the electronic search to include the highest number of articles. We did not conduct any research among patents.

### Data collection and analysis

The search results were screened by three reviewers (EB, MG, EA). The articles resulting from the database search were selected according to the following inclusion and exclusion criteria:

- The device must be soft at the target joint level, meaning that there should be no rigid links that impose physical constraints on joint motions. Devices containing some rigid parts elsewhere are included.
- The device must be intended to facilitate and support the movement of at least one degree of freedom of a joint among the shoulder, the elbow, or the wrist.
- The device must be intended to be wearable and portable (if it is not portable when described in the literature document, it must be meant to be in next iterations).
- The article is written in English and accessible as a full text by the Review Authors.

Hand devices were excluded from this review since they present particular design considerations and were already described by Chu and colleagues [18]. The Authors independently read the titles and the abstracts of retrieved articles and eliminated obviously irrelevant studies. The full texts of the remaining studies were examined and, according to the predetermined inclusion and exclusion criteria, they were independently ranked by at least two Authors as relevant and irrelevant. Discrepancies between Review Authors were resolved through discussion.

### Data extraction

To provide a comprehensive summary of the identified devices, we investigated the following features of the included studies:

- the intended application scenario, either rehabilitation, assistance, or augmentation, as previously classified;
- the actuation characteristics in terms of the type of actuator(s) used and the number and type of implemented degree(s) of freedom (DOF) or supported movements;
- the design approach followed with a focus on bio-inspired designs or other noteworthy design solutions;
- the implemented intention-detection strategies;
- the experiments conducted to validate the prototype.

The Technology Readiness Level (TRL) is also assessed for each device to provide a glimpse of the technological advancement in the field. The TRL is assigned according to the TRL definition provided by the HORIZON 2020 - Work Programme 2014-2015 and as explained in Table 2.

**Table 2:** TRL definition.

TRL	Definition	Explanation
1	Basic principle observed	The idea has been formulated
2	Technology concept formulated	The concept and the application have been formulated
3	Experimental proof of concept	The first prototype has been built but not tested
4	Technology validated in lab	The prototype has been tested in laboratory on a mannequin
5	Technology validated in relevant environment	The prototype is mature in terms of design and control and has been tested on healthy subjects
6	Technology demonstrated in relevant environment	The prototype has been tested for its efficacy on subjects with motor disability or healthy subjects according to the intended scenario
7	System prototype demonstration in operational environment	The system has been tested for its intended purpose in the clinic, at home, in the factory, in sport fields
8	System complete and qualified	The system is ready to be produced in large scale
9	Actual system proven in operational environment	The system is available in the market

## Results

### Results of the electronic search

**Fig. 1:**
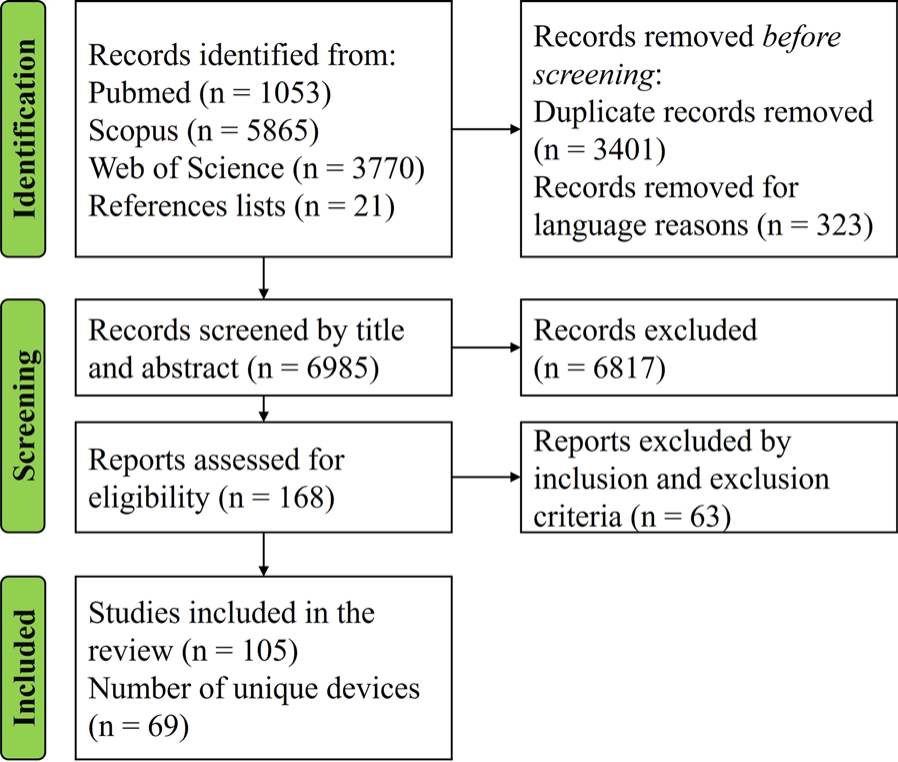
PRISMA flowchart of the literature search process.

A flow chart outlining the studies selection process is shown in Fig. 1.

The electronic databases’ search resulted in a total of 10688 identified studies. Searches through the reference lists resulted in 21 additional studies, reaching a total number of records of 10709. After the removal of 3401 duplicates and 323 articles that were not written in English, the titles and abstracts were pre-screened and irrelevant studies were eliminated. The full texts of 168 articles were analyzed and 105 articles, describing a total of 69 devices, were finally selected. Articles from the same research group were considered independently if the devices described were evidently different from each other, whereas articles regarding different iterations of the same device were grouped. For what concerns the design characteristics, the latest prototype iteration was considered, whereas, for what regards controllers, experiments, and evaluation metrics, the most representative across all papers concerning the same device were considered.

### Overview of the identified devices

**Fig. 2:**
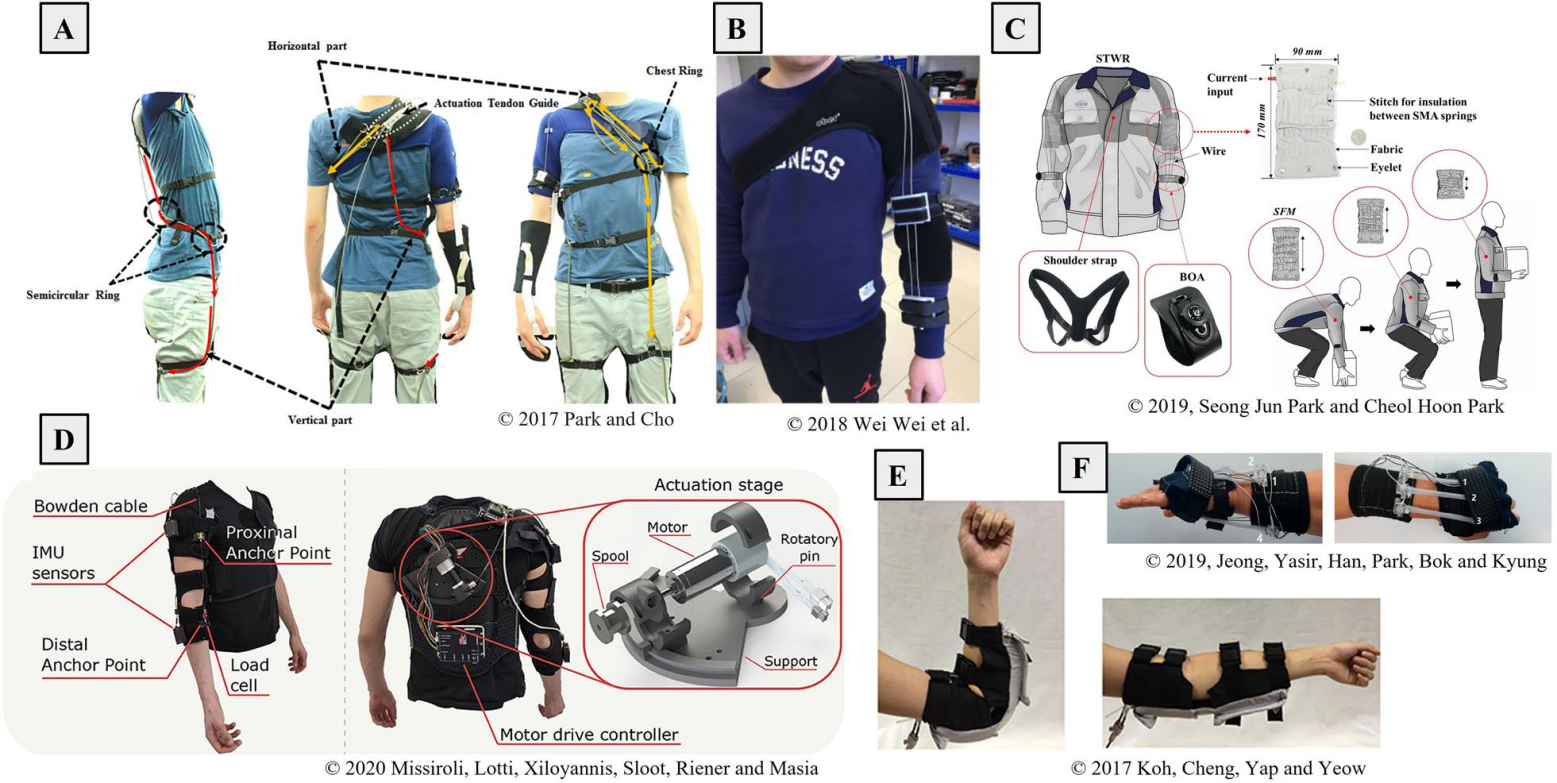
Examples of soft robotic wearable devices for the upper limb: **A** passive suit for the assistance of the shoulder elevation from [35], **B** cable-driven suit for the assistance of the elbow flexion-extension from [60], **C** shape memory alloy (SMA) suit for the assistance of elbows flexion-extension from [41], **D** cable-driven suit for the assistance of the elbow flexion-extension from [74] **E** Pneumatic sleeve for the assistance of the elbow flexion-extension from [24], **F** SMA glove for the assistance of the wrist flexion-extension and ulnar and radial deviation from [88]. All images are under the Creative Commons Attribution License, which permits unrestricted use, distribution, and reproduction in any medium, provided the original work is properly cited (https://creativecommons.org/licenses/by/4.0/).

An overview of the identified devices is provided in Tables 3, 4, 5, 6, 7, where information on the intended application, actuation (degrees of freedom and type of actuators) and control strategy is provided. The assigned TRL is also reported for each device. Tables are organized grouping the prototypes according to their actuation strategy (i.e., cable-driven, passive, pneumatic, shape memory alloy, hybrid). Examples of soft robotic wearable devices for the upper limb are shown in Fig. 2.

**Table 3:** Overview of the cable-driven devices.

Scenario	DOF	Low-level Control	High-level Control	TRL	Year and Related Works
R	1, S: abd/add	Position	–	4	2012 [58, 89]
R	1, E: flex/ext	-	–	3	2015 [54]
A	3, S: abd/add and flex/ext, E: flex/ext	Position	Mirroring the healthy limb	5	2017 [62]
R/A	7, S: humeral rot, abd/add, flex/ext, E: flex/ext, FA: pron/sup, wrist flex/ext and radial/ulnar dev	Position	Pre-determined joints trajectory	5	2018 [43, 90]
R	3, S: abd/add, flex/ext E: flex/ext	Speed	–	4	2018 [34]
A	1, E: flex/ext	Force	Arm dynamics compensation	4	2018 [60]
R	5, S: humeral rot, abd/add and flex/ext, E: flex/ext, FA: pron/sup	Position	Joystick or mirroring the healthy limb	6/7	2019 [55, 79, 91]
A	1, W: Dart throwing motion	Force	EMG-trigger	5	2019 [48]
A	2, E: flex/ext, FA: pron/sup	Position	–	4	2019 [92]
R	2, S: flex/ext, E: flex/ext	Position	Pre-determined joints trajectory	5	2019 [93]
R	1, E: flex/ext	Torque	Smartphone app. trigger	3	2019 [66]
R	1, E: flex/ext	Admittance	EMG-based neural-network torque estimation	5	2019 [56, 57, 94]
R/A/I	1, E: flex/ext	Admittance	IMU-based gravity compensation or EMG-based torque estimation	5/6	2020 [28–32, 53, 74, 78, 95–101]
I	2: bimanual lifting	Force	EMG trigger	5/6	2020 [73, 102]
I	4, S: elev, E: flex, bilateral	Position	Voice recognition	5/6	2020 [52, 103]
I	1, E: flex/ext	-	–	3	2020 [59]
R	1,W: flex/ext, ulnar/radial dev	Position	Pre-determined joints trajectory	6	2020 [104]
-	2, S: elev, E: flex/ext	Position	Pre-determined joints trajectory	5/6	2020 [61, 105, 106]
A	1, S: elev coupled with hum rot	Admittance	Gravity compensation	5	2020 [33]
R	1, E: flex/ext	Motion	-	4	2020 [107]

S = shoulder, E = elbow, FA = forearm, W = wrist, abd = abduction, add = adduction, flex = flexion, ext = extension, rot = rotation, pron = pronation, sup = supination, dev = deviation, elev = elevation, R = rehabilitation, A = assistance, I = industrial

**Table 4:** Overview of the passive devices.

Scenario	DOF	Low-level Control	High-level Control	TRL	Year and Related Works
I	1, S: elev	–	–	6	2017 [35]
I	1, Weight lifting support	–	–	4	2018 [36]
A	1, E: flex/ext	–	–	5	2019 [38]
A	1, S: elev	-	–	4	2020 [37]

S = shoulder, E = elbow, flex = flexion, ext = extension, elev = elevation, A = assistance, I = industrial

**Table 5:** Overview of the pneumatic devices.

Scenario	DOF	Low-level Control	High-level Control	TRL	Year and Related works
A	2, S: abd/add, E: flex/ext	Pressure	–	4	2004 [108, 109]
A	1, W: flex/ext	Pressure	Bending signal trigger	5	2005 [25]
R	4, E: flex/ext, FA: pron/sup, W: flex/ext, ulnar/radial dev	Force	Individual actuators control to induce muscle activation pattern	5	2013 [110–115]
R	3, FA: pron/sup, W: flex/ext, ulnar/radial dev	Pressure	–	4/5	2015 [116]
R	2, W: flex/ext radial/ulnar dev	Position	–	5	2015 [76]
R	1, E: flex/ext	Position	Pre-defined joint trajectory	5	2015 [22]
A	1, S: abd/add	Position	–	4	2016 [15]
R	2, W: flex/ext, radial/ulnar dev	Pressure	Manually defined setpoint	4	2017 [117, 118]
R	1, E: flex/ext	Pressure	Pre-defined or EMG triggered	5	2017 [24]
A	2, S: abd/add, flex/ext	Position	–	4	2017 [14]
A	1, S: abd/add	Position	–	4	2017 [119]
R	3, S: flex-ext, E: flex-ext, FA: pron-sup	Pressure	Manually defined setpoint	4	2018 [26]
A/I	1, E: flex/ext	-	–	4	2018 [120, 121]
A	1, E: flex/ext	Pressure	Bending signal trigger	5	2018 [122, 123]
Sport	1, S: Bat swing assistance in baseball	Pressure	Acceleration signal trigger	5	2018 [50]
Sport	1, S: forehand swing motion Medical	Pressure	Electric valve switch	5	2018 [51]
R	2, E: flex/ext, FA: pron/sup	Pressure	–	4	2018 [124]
R	2, W: flex/ext, radial/ulnar dev	Position	Mirroring the healthy limb	5	2018 [70]
R	2, S: Bimanual wheelchair push	Pressure	IMUs signal-based trigger	5	2018 [69]
I	1, E: flex/ext	Pressure	Manually defined setpoint	4	2018 [20]
Medical	3, S: abd/add, flex/ext, E: flex/ext	Pressure	–	4	2019 [77, 125]
-	1, E: flex/ext	Pressure	–	4	2019 [126]
R/A	2, S: abd/add, flex/ext	Pressure	Joystick	4	2019 [21]
R	1, S: abd/add	Pressure	Buttons trigger inflation and deflation	4/5	2019 [63]
R	1, E: flex/ext	Position	Pre-defined joint trajectory	3	2019 [23, 127]
R	2, W: flex/ext, radial/ulnar dev	Pressure	–	4	2019 [128]
R	1, FA: pron/sup	Position	Pre-defined joint trajectory	4	2019 [129]
R	2, S: elev, E: flex/ext	Position	Mirroring the healthy limb	5	2019 [71]
R	1, FA: pron/sup	Pressure	Pre-defined assistance levels	4	2019 [130]
R	1, S: abd/add	Pressure	Linear increase of pressure during abduction	4	2019 [131]
R	4, S: flex/ext, E: flex/ext, FA: pron/sup, W: flex/ext	Pressure	Button-triggered manually adjustable setpoint	5/6	2020 [64, 132]
I/R	3, S: flex/ext, E: flex/ext, W: flex/ext both single and dual arm	Force	Action and pose recognition	6	2020 [65, 67]
R/A	1	Pressure	–	4	2020 [133]
R	1, S: abd/add	-	–	3	2020 [27]
Medical	1, S: abd/add	-	–	4	2020 [134]
R	1, S: elev	Pressure	Manually defined setpoint	5/6	2020 [19]

S = shoulder, E = elbow, FA = forearm, W = wrist, abd = abduction, add = adduction, flex = flexion, ext = extension, rot = rotation, pron = pronation, sup = supination, dev = deviation, elev = elevation, R = rehabilitation, A = assistance, I = industrial

**Table 6:** Overview of the SMA devices.

Scenario	DOF	Low-level Control	High-level Control	TRL	Year and Related works
R	1, W: flex/ext	Position-velocity	–	3	2015 [39]
R	1, E: flex/ext	Position	Pre-defined joint trajectory	5	2017 [40]
R	2, W: flex/ext, ulnar/radial dev	Temperature	–	4	2019 [88]
-	1, E: flex/ext	Position	Pre-defined joint trajectory	4	2019 [41]

E = elbow, W = wrist, flex = flexion, ext = extension, dev = deviation, R = rehabilitation

**Table 7:** Overview of the spring-blades and hybrid devices.

Scenario	DOF	Low-level Control	High-level Control	TRL	Year and Related works
R	2, W: flex/ext, ulnar/radial dev	Motion	Buttons-triggered	4	2018 [42]
R	2, W: flex/ext, radial/ulnar dev	Position	EEG-based attention trigger	5	2019 [72]
A	2, S: flex/ext, E: flex/ext	Position	Reed switches trigger forward and backward driving	5	2015 [46, 47]
I	1, S: elev	Pressure	–	4	2019 [44]
R	2, E: flex/ext, W: flex/ext	Pulse-width (NMES), pressure	EMG trigger	5/6	2020 [45]

S = shoulder, E = elbow, W = wrist, flex = flexion, ext = extension, dev = deviation, elev = elevation, R = rehabilitation, A= assistance, I = industrial

### Application scenario

The first step in the process of designing a device to support limb movements is the definition of the intended application. This is fundamental since it influences all the subsequent design choices: types and number of actuators, materials, intention detection strategy, and controller architecture.

**Fig. 3:**
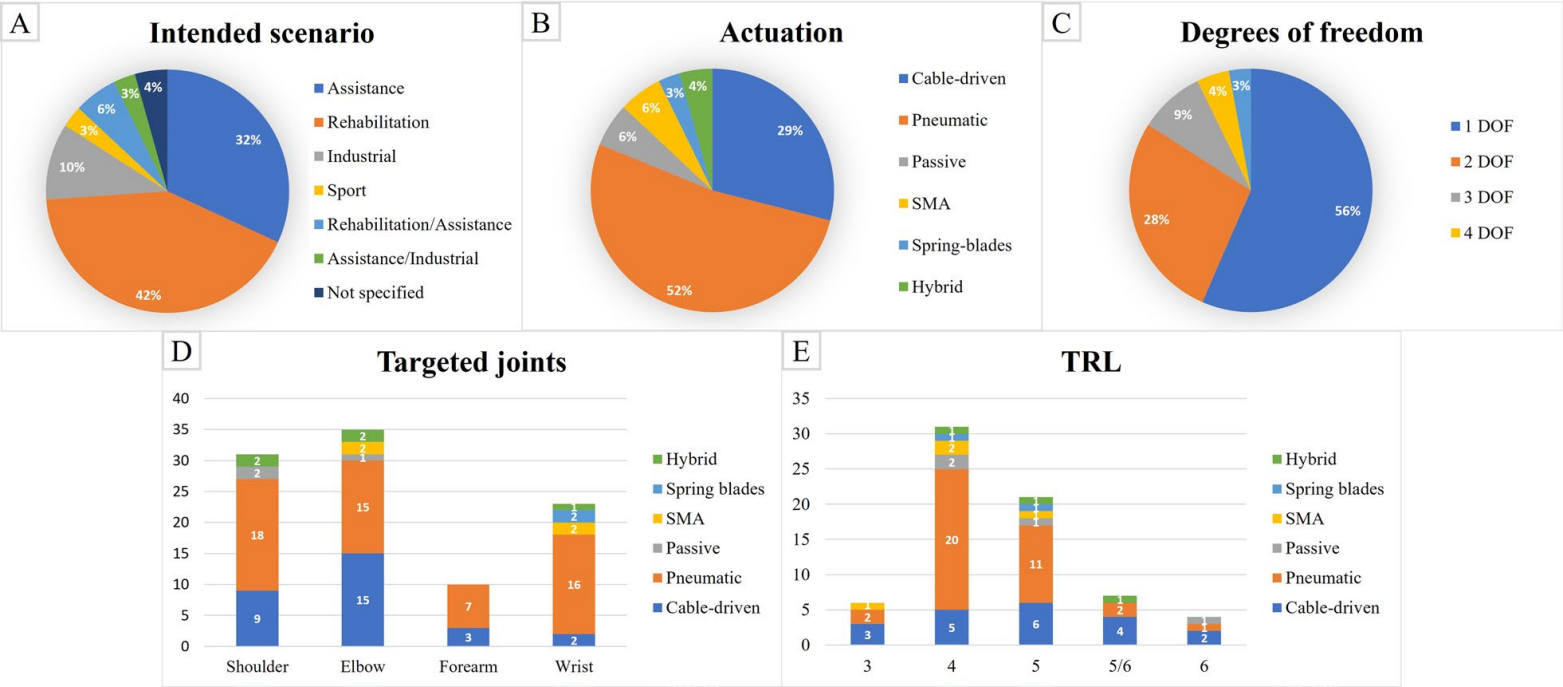
Results of the electronic search in terms of **A** intended application scenario of the device, **B** actuation type, **C** implemented degrees of freedom, **D** joints actuated by the device with respect to the actuation type, **E** technology readiness level with respect to the actuation type, where devices scoring half-levels were grouped with the lowest level, except for category 5/6 which was numerous.

The three possible scenarios we identified in the selected works are: (i) rehabilitation, (ii) assistance with ADLs, (iii) augmentation of healthy subjects in environments such as industry or sport. The majority of devices (81%) are meant for medical applications, either for assistance (32%) or rehabilitation (42%) or both (6%), as shown in Fig. 3A.

The low number of augmentation devices for healthy subjects, either for industrial (13%) or sport (3%) applications, is probably because soft robots are not the best choice to provide the forces required to carry out arduous work. Indeed, the fact that they rely on the skeletal structure of the wearer to transfer the forces prevents them to apply high torques. Moreover, their intrinsic compliance does not guarantee precision in trajectory tracking. On the other hand, the compliance of such devices is the key feature to guarantee comfort and safety, thus making them good candidates to provide prolonged assistance.

### Actuation

The second step to be accomplished when designing a soft exoskeleton is the choice of the actuators. Each actuation type has its own advantages and disadvantages, thus the selection should be made according to the requirements of the device, possibly based on the application scenario. Moreover, it should be considered that the device is meant to be portable and, therefore, the shape and the weight become critical features. The actuation types we identified in the literature analysis are pneumatic (52%), cable-driven (29%), passive (6%), Shape Memory Alloy (SMA) (6%), spring blades with linear actuators (3%), and hybrid actuation systems (4%), as shown in Fig. 3B.

Despite the characteristics and the pros and cons of the different types of actuators were deeply discussed by Thalman and colleagues [17], we will provide a brief summary hereafter for completeness.

#### Pneumatic actuation

Pneumatic actuators use compressed air to actuate a system. An example of a pneumatic suit is shown in Fig.2E. They can contract, expand, elongate, and even bend upon inflation. They are compliant and can be placed along the limb to distribute the contact pressure [19–22]. The main disadvantages of pneumatic actuation lie in the low bandwidth and in the non-linearity [23–27]. Moreover, they need compressors and tanks, which impact the portability of the system.

#### Cable-driven actuation

Cable-driven transmission relies on the use of cables that are wound up on a spool by the action of electric motors. Examples of cable-driven exosuits are shown in Fig. 2E and D. The shortening of the cables produces a positive tension that acts on the anchor points. The use of electric motors brings advantages such as ease of control with respect to other actuators, higher power, and higher bandwidth. Cables are inserted into sheaths (Bowden cables) to reduce friction and protect the wearer. Indeed, cable transmission presents disadvantages in terms of friction and backlash phenomena [28, 29] which must be considered when designing the suit and the control architecture [30–34].

#### Passive systems

Three types of passive mechanisms were identified among the selected devices: (i) spring and cable transmission [35–37], (ii) locking mechanisms [36], (iii) elastic bands [38]. An example of a passive exosuit is shown in Fig. 2A. The advantage of using passive systems is that they do not require any source of power, thus reducing the weight of the device, solving battery duration issues, and being cost-effective [37]. The disadvantage is that they may only provide either gravity compensation or help to maintain a posture, without being able to adapt to external conditions.

#### Shape Memory Alloy actuation

Shape Memory Alloys (SMA) can be used in those applications where small and slow movements are required due to their limited stroke and bandwidth [39]. Examples of SMA eoxsuit are shown in Fig. 2C and F. They have a low weight and small dimensions, they are noiseless, and are relatively cheap [40, 41]. However, they present a highly non-linear behavior due to hysteresis and their control depends on room temperature [40].

#### Spring blades actuation

This type of actuation is based on the use of linear electric motors and elastic metallic strips. The linear motors pull or push the metallic blades which actuate the joint. The actuation unit is placed directly on the limb which needs support [42, 43]. This is advantageous in terms of transmission but increases the weight of the arm, at the expense of portability.

#### Hybrid actuation

Hybrid systems combine different types of actuators in a single device. One device combines pneumatic actuators and cable-driven transmission [44]. The authors claim that this solution combines the strength and compliance of the pneumatic actuators, with the convenience of the Bowden transmission. In another device, pneumatic actuators and neuromuscular electrical stimulation (NMES) were combined to boost the rehabilitative potential of the device [45]. Finally, one device includes passive springs in an active cable-driven device to reduce the motor power consumption [46, 47]. The authors proved that the passive spring system decreased the motor power consumption by 17.5%.

### Actuated joints

The majority of the identified devices actuate only one (56%) or two (28%) DOFs, as shown in Fig. 3C. Few works have actuated more than two DOFs. This may be because a large number of actuators is required to actuate multiple DOFs, which could in turn make the device cumbersome and heavy, while also making the control challenging.

Among the human arm joints, the shoulder is actuated in 45% of the identified devices, the elbow in 51%, the forearm in 10%, and the wrist in 33%. In Fig. 3D, the number of devices actuating each joint with respect to the type of actuation is shown.

With regards to shoulder actuation, it was mainly implemented with pneumatic actuators (58%). The actuation of the elbow is instead equally distributed between pneumatic actuation and cable-driven transmission (43%). The forearm pronation/supination and the wrist movements are mainly supported by pneumatic actuators (70%).

Some research has been undertaken to combine multiple DOFs by exploiting arm kinematic couplings to simplify the design. Choi and colleagues [48] implemented the Dart Throwing Motion for the wrist, whereas Georgarakis and co-workers [49] proposed the coupled elevation and external rotation of the shoulder in the frontal plane. Other combined movements that were implemented are the bat swing assistance for baseball augmentation [50] and the forehand swing assistance for tennis augmentation [51].

Similarly, attempts were conducted to actuate different DOFs in series. Kim and colleagues [52] implemented a locking mechanism, which allows actuating both elbow and shoulder in consecutive phases. Xiloyannis and co-workers [53] developed a modular one-to-many actuator, which allows for the independent control of two elbows with a single actuator.

### Design approach

To improve the design phase, some works undertook a “bio-inspired” approach, taking inspiration from the human musculoskeletal structure to design the device.

In the case of cable-driven devices, cable routing can be bio-inspired in the sense that it mimics muscle insertions and tendon positions. Moreover, cable guides can reproduce the function of important skeletal structures. An example is the elbow cable guide, which can be used to mimic the olecranon function, which acts as a lever for the extensor muscle [34, 54]. A different approach used to perform cable routing is the identification of lines of minimal extension to place rigid parts such as cable guides and Bowden sheaths, as was proposed by Lessard et al. [55]. Lines of minimal extension are defined as those where the skin shears less.

Similarly, pneumatic actuators can be placed in the same position as the target muscles in a bio-inspired fashion. Moreover, pneumatic actuators could be shaped to fit the curvature of the arm, as in [20]. In analogy with what can be done for cable-driven systems, pneumatic devices can include mechanisms that mimic the musculoskeletal structure. An example is given by O’Neill and colleagues [14], who mimicked the shoulder cruciate ligament with a four-bar linkage system.

An example of a bio-inspired approach in passive systems is the one proposed by Phan and co-workers [38], who placed the passive elastic bands so as to emulate the ligaments’ structure of the elbow. Bio-inspiration may also apply to the design of the actuator itself. Indeed, a cable-driven compliant tendon sheath actuator based on the Hill muscle-tendon model was designed by Lu and colleagues [56, 57].

In addition to the bio-inspired approaches, other significant design solutions, which are aimed at improving ergonomics and energy-saving, have been proposed in the literature.

Galiana and colleagues [58] used redundant collinear cables to solve misalignment problems and to reduce off-axis torques of the shoulder. They proposed to insert the cables anchor points in such a way to minimize the motor torque required to perform the movement and to minimize the non-targeted joints torques. The goal was to achieve transparency with respect to the non-assisted DOFs, bringing advantages both in terms of energy savings and ergonomics. Another approach to deal with alignment problems has been proposed by Harbauer and colleagues [59]. They secured the cable at the wrist in a loop, so that in case there is a slight offset between the motors or the upper arm is rotated, it can be compensated. This approach also allows an even distribution of forces between both sides of the arm to be achieved.

For what concerns pressure distribution on the limb, Wei and colleagues [60] increased the number of Bowden cables from the minimum necessary to reduce pressure. Samper-Escudero and co-workers [61] proposed an exosuit coupling system based on fibers compliance: the textile pattern was designed to efficiently transmit forces. The coupling transforms the pulling force from the cable into a pushing force over the anterior part of the limb. When the cable tightens, the clamp adheres to the anterior part of the arm, creating a push-force in the area, and the fabric self-adapts to the user’s anatomy.

Park and co-workers [35] were able to decouple the horizontal movement of the shoulder from the vertical movement by using a hook tendon, which is free to move from the anterior to the medial part of the arm. This strategy allows the actuation tendon to only slightly change length when the horizontal movement is performed, so that elevation of the arm can be effectively assisted in any configuration. Moreover, the hook detaches when the applied torque exceeds 10% of the maximum torque required. Something similar was done by Kim and colleagues [52], who introduced a rotating anchor point above the shoulder to allow for passive internal/external rotation of the shoulder.

To relieve compression forces and shear stresses from the shoulder, Gaponov and colleagues [62] and Li and colleagues [34] applied an offset to the shoulder.

To reduce the weight of the air supply in their pneumatic actuated tennis augmentation device, Ogawa and colleagues [51] exploited the weight of the tennis player to supply pressure from a pump placed in his/her shoes.

### Intention detection

Among the 69 identified works, only 23 implement an intention detection strategy. In the next paragraphs, the solutions adopted are briefly described. The remaining works either defined a trajectory to be tracked or manually adjusted the support provided by the suit.

#### Switches, buttons, joysticks

As for electric motors, buttons and/or switches can be used to trigger forward and backward driving of the actuators [42, 46, 47, 51]. The motors are usually actuated at a constant speed and the switches are used to control position. In the case of pneumatic actuators, instead, one button can be employed for inflation and another for deflation [63–65].

Similarly, a joystick [21, 55] can be used to perform position control of the limb. Seth and colleagues [66] implemented a smartphone application to be used as a touch trigger, which initiates flexion and extension at constant torque.

The main drawback of all these approaches lies in the need to use the controlateral arm to trigger the assistance. Therefore, this strategy interferes with the normal execution of ADLs, requires some residual capability of the controlateral arm, and can assist only mono-lateral movements.

An approach that would solve this problem is voice recognition [52]. However, this type of strategy can be used in almost noise-free environments only.

#### Kinematic and dynamic measurements

If the user is healthy or she/he has residual motor capabilities, it is possible to implement IMU-based torque estimation. The control action can be computed by estimating the torque necessary to compensate for gravity [33, 56, 67, 68] or by implementing a dynamic torque estimator [30], which takes also the dynamics of the arm into account.

IMUs can also be used to trigger assistance when the acceleration signals overcome a threshold [50] or to detect the phase of cyclic movements, such as pushing the wheelchair, and to provide assistance in the appropriate phase [69].

Similarly, it is possible to exploit the signal coming from bending sensors [25].

Another popular approach is mimetic control, or mirror position control [55, 62, 70, 71]. Assuming that one of the two arms is healthy, it is possible to track its movements and reproduce them on the impaired limb thanks to the device. This technique, however, only allows the suit to provide support with bimanual and symmetric tasks.

#### EEG measurements

To make sure the patient is highly involved in the training, Li et al. [72] implemented assistance triggered by measuring the electroencephalography (EEG) attention level. The amplitude of the wrist movement was pre-determined. Attention levels were calculated according to the EEG band power values.

#### EMG measurements

In case the subject preserved some residual muscular activity, surface electromyographic (sEMG) signals can be used to trigger the assistance. Different modalities have been implemented. The easiest way to use it is by imposing a threshold that the signal must overcome to trigger assistance. The threshold can be single [45, 48] or double [73]. It is also possible to impose a time-over-threshold parameter which is useful to reject artifacts [24].

EMG signals can be used to estimate the muscle torque [57, 74], thus determining the level of assistance. The advantage of this approach is that the user’s intention is detected before the actual execution of the movement, and therefore the assistance is provided in a timely manner [75]. Moreover, using EMG signals allows the monitoring of the user’s active involvement in the execution of the task, improving rehabilitation outcomes. The main drawback of using such strategies is the need for performing a calibration procedure for each user and each session, due to the replacement of the electrodes.

Lotti and colleagues [32] made a comparison between IMU-based and EMG-based assistance. They concluded that the two strategies show similar performances in terms of position tracking accuracy and muscular effort reduction. However, EMG-based intention detection is capable of adapting when dynamic conditions change, thus making the use of the device more symbiotic with respect to the IMU-based approach.

### Validation

To test the efficacy of the device in supporting the user’s motion, validation tests were conducted on most of the identified devices. In particular, 7 devices were tested on subjects with disability (either post-stroke or elderly), 50 devices were tested on healthy subjects, and the remaining were either tested on test benches or on a mannequin. Experiments carried out solely to evaluate the performance of the actuators will not be described. The validation tests identified in this literature review can be grouped into four main categories: i) kinematics evaluation, ii) user’s effort evaluation, iii) comfort and ergonomics evaluations, iv) clinical assessment.

#### Kinematics evaluation

Concerning kinematics, the main outcome measures are trajectory tracking performance, range of motion (ROM), and movement execution velocity.

Trajectory tracking performance tests are conducted in case the control strategy relies on a reference trajectory to evaluate whether the suit can achieve the reference movement and with which accuracy.

For what regards cable-driven devices, Gaponov and co-workers [62] tested a possible rehabilitation scenario on four healthy subjects in which mirror therapy was delivered. They obtained an angle root mean squared error (RMSE) of 2.12$^\circ$, 3.05$^\circ$, 4.9$^\circ$ for the shoulder flexion, shoulder abduction, and elbow flexion, respectively. Li and colleagues [34] tested their device on healthy subjects and obtained an angle RMSE of 2.3$^\circ$ and of 2.9$^\circ$ for the shoulder movement and the elbow flexion, respectively. Choi and colleagues [48] achieved an angle RMSE lower than 3$^\circ$ for the wrist dart-throwing motion of three healthy subjects. With respect to pneumatic devices, Andrikopoulos and colleagues [76] achieved mean absolute errors of 1.43$^\circ$ and 1.51$^\circ$ for the flexion/extension and radial/ulnar deviation of the wrist, respectively, for one healthy subject.

When the range of motion is tested, this is mainly done in three different conditions: i) without the suit, to understand the natural ROM of the user, ii) with the suit but without assistance, to understand the effects of the suit design, iii) and with the suit and assistance provided, to understand the augmentation properties of the device.

To cite a few examples, Choi and colleagues [48] achieved a ROM higher than 50$^\circ$ for the wrist flexion of one healthy subject. They also proved that their wrist-assistive device did not influence the ROM of non-targeted joints, such as the fingers, obtaining no significant change with respect to the natural ROM. Similarly, Lessard and colleagues [55] proved that their prototype does not significantly influence the ROM of healthy subjects. Koh and co-workers’ elbow sleeve [24], instead, achieved approximately 50% of the active ROM when no contribution was provided by the healthy user.

The velocity of the movement was evaluated by Sakoda and colleagues [50] who achieved an improvement in bat swing speed by 3 km/h in experienced subjects. Similarly, Ogawa and co-workers [51] proved that their suit could significantly improve tennis swing velocity in healthy subjects. Xiloyannis and colleagues [28] instead observed that the use of the exosuit decreases the movement velocity and smoothness in healthy subjects.

#### User’s effort evaluation

The effort of the user in performing a certain movement is evaluated to test the efficacy of the device in providing support. Typically, sEMG signals are evaluated while performing dynamic or static tasks with different loads, both on targeted and non-targeted muscles. Comparisons are made when performing the task without wearing the device, wearing it but without assistance, and with assistance. Isometric tests are also conducted to assess the effect of the suit on muscle fatigue. Xiloyannis and co-workers provided a nice overview of the results obtained by different research groups in terms of sEMG signal in their recent review article [11]. In general, exosuits prove to be effective in decreasing muscular effort.

Another option to test muscular endurance with and without assistance is explored by Sasaki and co-workers [25] with Mosso’s ergograph, obtaining a clear improvement in muscular endurance for healthy subjects. The measurement of body sway is instead used as an indicator of muscle fatigue by Abe and colleagues [77], whose suit was could suppress body sway by 5% for one healthy subject. The maximum load holding time is exploited by Goppold and colleagues [65] to test the ability of their device to extend it. The maximum holding time was extended by approximately 50%, when support was provided by the suit to six healthy subjects.

An alternative to assess the user’s effort is to estimate the metabolic consumption from the heart rate (HR), as done by Lessard and colleagues [55]. They proved that the suit could reduce the HR increase of subjects with single-arm weakness, when exercising, from 12.4% to 3%. O’Neill and colleagues [19] measured the change in HR of the therapist when delivering stretching exercises with and without the assistance of the suit and obtained a decrease between 3.2% and 8.6%.

#### Comfort and ergonomics evaluation

Regarding comfort and ergonomics, this is evaluated by measuring contact forces between the limb and the device, as done by Andrikopoulos and colleagues [76] while performing flexion-extension and ulnar-radial deviation of the elbow. The forces generated by the device on the hand and palm did not exceed 2.2 N. Similarly, Xiloyannis and co-workers [78] evaluated the pressure distribution at the human-suit interface of the anchor point achieving peak pressure ranging from 20 to 40kPa.

Other methods of assessing comfort are checking skin redness [19] or providing questionnaires directly to the user for a self-evaluation [65].

#### Clinical assessment

Only one study [45] evaluated the effect of using a hybrid exosuit, integrating NMES and soft pneumatic muscle, on upper limb motor recovery of a group of 15 stroke survivors. The study showed that a 20-session training significantly improved voluntary motor functions, released muscle spasticity at the elbow, wrist, and fingers, and improved muscular coordination of the entire upper limb.

### Technology Readiness Level

As can be observed in Fig. 3E, most of the devices have a TRL of 4 or 5. In particular, 9% of the devices score level 3, 45% score level 4, 30% score level 5, 10% were assigned between level 5 and level 6, 6% score level 6. The majority of pneumatic devices (55%) score level 4, whereas the majority of cable-driven devices (33%) score level 5.

Level 3 means that the experimental proof of concept or the prototype has been built but not tested. Level 4 means that the prototype has been tested in the laboratory on a mannequin. Level 5 means that the prototype is mature in terms of design and control and has been tested on healthy subjects. Level 6 means that the prototype has been tested for its efficacy on subjects with motor disabilities or healthy subjects according to the intended scenario. Level 7 means that the system has been tested for its intended purpose in the clinic, at home, in the factory, or in sports fields.

Few devices reached maturity, in terms of design, control, and safety, for being tested on end-users (level 6).

Among the 9 devices with the highest TRL (5/6-7), only three actuate more than 2 DOFs. The first one is a cable-driven device developed by Lessard and co-workers [79], it actuates 6 DOFs and was tested in a clinical environment on 9 subjects with upper limb impairment. However, it only supports symmetric tasks, since it relies on a mimetic control strategy. The second one, a pneumatic exosuit, was developed by Das and co-workers [64]. It supports 4 DOFs but, at the current state, it is not portable and the support is changed by employing a potentiometer, which makes the support not dynamic. The third one, a pneumatically actuated exosuit, was designed by Goppold and colleagues [65]. It actuates 3 DOFs and automatically recognizes the action and the pose of the user. However, some critical aspects were found to be the high weight (6.5 kg) and the low intuitiveness of the device to be worn and used.

The exosuit designed by Samper-Escudero and co-workers [61], supports 2 DOFs (shoulder elevation and elbow flexion) but relies on pre-determined joint trajectories. The device developed by Xiloyannis and colleagues [28, 74] instead was used with a variety of intuitive controllers that do not require the user’s input (EMG-based torque estimation or IMU-based gravity compensation) but only supports the elbow. Similarly, the exosuit developed by Hosseini and colleagues [73] implements an EMG-based controller, but only supports bimanual lifting. Another exosuit meant for industrial work, developed by Kim and co-workers [52], supports bimanual lifting, with shoulder elevation and elbow flexion coupled, with the assistance activated via voice recognition.

Finally, O’Neill and colleagues [19] developed a pneumatic exosuit to support the patient’s arm against gravity so as to relieve the therapist’s burden during the exercise. However, the support level is manually adjusted according to the user’s and therapist’s needs.

## Discussion

The results of this review show that research in the field of upper limb soft devices has been quite active in the last two decades. The main applications for which they have been developed are related to the assistance of fragile people, either for rehabilitation, or assistance, or both. Cable-driven transmission and soft pneumatic actuators are the most common choices for the actuation unit.

In particular, pneumatic actuators seem to be the preferred choice to actuate the shoulder. This may be because to actuate the shoulder one must deal with the whole weight of the arm. Indeed, they may be placed below the arm and inflated to push the arm, evenly distributing contact pressure. The use of cable-driven transmission might be sub-optimal since it could result in pressure peaks around the anchor points. Spring blades and SMA may not be indicated due to the relatively limited stroke they are capable of, making them more suitable for supporting wrist movements.

Nevertheless, research on soft pneumatic actuated devices is still mainly focused on the development of the actuators themselves, whereas cable-driven devices can be considered more advanced in terms of control strategies and intention detection.

The assistance of the elbow joint is the most explored, followed by the shoulder and the wrist. Few works have implemented more than 2 DOFs, due to the complexity of the control. Those who have, indeed, either do not implement advanced controllers, including intention-detection strategies appropriate for the intended scenario, or did not test a great variety of movements.

Regarding design solutions, even though many attempts were conducted to improve ergonomics and energy saving, we believe there is still scope for improvement. Effort should be put into designing the suit to optimize pressure distribution and joint reaction forces. Simulation software, such as OpenSim (SimTK) [80, 81], could be a useful resource in this view. Moreover, few works have achieved real portability at the latest iteration.

A common problem in soft devices is the inability to apply high forces because of the deformability of the soft materials and the fact that the device relies on the skeletal structure of the user. Optimization of the comfort and ergonomics should go in parallel with the improvements in force transmission. Research should also be devoted to making the devices adaptable to different users according to the their physical characteristics.

We believe that the integration of passive systems such as springs, clutches, and brakes in active devices could be beneficial in the decrease of energy consumption and weight of the device. Although only preliminary results were found in this review, this suggestion can be supported by literature on upper limb stiff exoskeletons that include passive elements in their design [9, 82, 83].

Hybrid actuation should be better explored as well, since it may allow the combination of the benefits of different actuation types, also according to the different joints to be supported. This might apply, in particular, to NMES hybrid devices. Indeed, NMES used in combination with rigid exoskeletons has proven to bring beneficial effects in upper-limb rehabilitation [84, 85]. We believe that, starting from the work of Nam and colleagues [45], this approach should be investigated with soft robotic suits as well.

Few prototypes implement intention detection strategies, which make the device effectively cooperate with the user. This, however, is a fundamental aspect of a device for motion support, especially if it is meant to assist with ADLs or arduous work. The development of effective intention detection strategies, which do not require long calibration procedures, and which are able to adapt to different dynamic conditions, is fundamental in view of the development and diffusion of soft devices for the upper limb, whose movements are various and hardly predictable.

Many works tested the device at a preliminary stage (actuation characterization), but few tests were conducted to assess the efficacy of the support. Experiments were conducted mainly to evaluate the accuracy of the device in tracking a certain trajectory, achieving full range of motion, and being effective in decreasing muscle effort when worn. However, proposed experimental protocols are various and differ from each other so that comparison among different works is difficult.

Few research groups tested their devices on end-users and performed a complete meaningful clinical assessment. For the rehabilitation scenario, in the future, other metrics commonly used for the evaluation of upper limb robotic devices such as movement time, hand-path ratio, and inter-joint correlation, should be included [86, 87]. In the assistance scenario, externally-assessed functional scales, such as the Performance of the Upper Limb-PUL-module, self-perceived scales, such as the Abilhand questionnaire, and usability scales, such as the System Usability Scale should be used to assess the effectiveness and usability of the device [3].

In this view, carefully designed pilot clinical trials should be conducted to prove the effectiveness of such devices to provide support to the wearer. The lack of clinical trials has also prevented to identify a precise target population.

It is worth noticing that most cable-driven devices score level 5, whereas most pneumatic devices score level 4. This suggests that cable-driven devices are more technologically advanced with respect to pneumatic ones. Moreover, the devices showing the highest TRL, either support only one DOF or implement control strategies that make them unsuitable for daily assistance with different activities (e.g. predefined trajectories, mimetic control, manual support adjustment, voice control). In the future, we would expect more suits to reach levels 6 and 7 and finally be ready for commercialization. Clinical trials will be also mandatory to prove that the devices meet the safety, health, and environmental protection requirements needed to reach the market.

## Conclusion

In conclusion, we confirm that soft exoskeletons might show a very high potential despite none of the analyzed devices having reached the market yet. We could infer that there is a lack of soft devices implementing multiple DOFs with appropriate control strategies including intention detection. The movements supported by the prototypes are few and do not include a great variety of actions that would allow the user to perform daily life activities. Ergonomics, user experience, and portability should be further explored in future studies, as well as the design of devices that support multiple DOFs. Finally, clinical trials are needed to assess the effectiveness of upper limb exosuits and to identify the category of subjects who can benefit the most from these types of devices.

Overall, we think that the development of soft wearable devices for the support of daily life activities of the upper limbs is a promising and growing research field for which we have great hopes in the next years.

## Data Availability

The dataset supporting the conclusions of this article is included within the article.

## Abbreviations

ADL: Ativities of daily living
DOF: Degree of freedom
EEG: Electroencephalography
HR: Heart rate
IMU: Inertial measurement unit
RMSE: Root mean square error
ROM: Range of motion
sEMG: Surface electromyography
SMA: Shape memory alloy
TRL: Technology readiness level
NMES: Neuromuscular electrical stimulation
